# RideGuide: multimodal conversational tour guide for passenger engagement and spatial learning in robotaxis

**DOI:** 10.1007/s12193-026-00479-2

**Published:** 2026-08-03

**Authors:** Eve Schade

**Affiliations:** University of St. Gallen, St. Gallen, Switzerland

**Keywords:** Autonomous vehicles, Multimodal conversational user interface, Large language models, Passenger engagement, Spatial learning, Robotaxi

## Abstract

Robotaxis are gradually taking the place of traditional taxis, removing the human driver as a source of local insights and conversation, fostering passive travel and potentially hindering passengers’ spatial learning of their surroundings. This paper introduces RideGuide, a customizable multimodal conversational tour guide system for autonomous vehicles that integrates voice, touch, and vision capabilities to provide context-aware and engaging interactions. In an exploratory lab study (*n* = 12), participants customized RideGuide ’s language, voice, and chatbot personality before experiencing a pre-recorded robotaxi ride (t = 10 min, 270$^\circ$ car back-seat video). Results indicate a positive hedonic user experience (UEQ-S, 1.10), moderate chatbot usability (CUQ, 67.6%), and low workload (NASA-TLX, 32.4%). On average, participants recalled 3.3 landmarks by name, suggesting a potential in supporting spatial learning. Participants reported a greater willingness to converse with RideGuide compared to human drivers and expressed openness to data sharing for personalization. The results show expectations for future robotaxi interfaces, including real-time contextual information, vehicle explainability, and adaptable conversation styles. The findings inform design recommendations for developing engaging, human-centered multimodal interfaces in autonomous mobility contexts.

## Introduction

Robotaxis are gradually taking the place of traditional taxis, altering passenger experiences by removing the informal interactions once provided by human drivers. Companies such as Waymo One and Baidu are rapidly expanding autonomous ride-hailing services in their corresponding markets [1, 2], with projections of wide accessibility of robotaxis in the commercial market by 2030 [3, 4]. While factors like affordability, efficiency, safety, and novelty are driving adoption [5–7], autonomous tourism and everyday urban mobility are also expected to gain traction  [5, 8, 9]. In traditional taxis, passengers can chat with the driver to ask about local recommendations or to engage in small talk [6, 10]. In contrast, without a human driver, robotaxi passengers are expected to occupy themselves with unrelated activities, shifting from an active to a passive travel experience [8, 11, 12]. Since autonomous vehicle (AV) passengers engage less with their surroundings, their spatial knowledge acquisition can be impaired, as early evidence suggest [13], mirroring effects observed among navigation app users [14–21].

Research in conversational user interfaces (CUIs) and advances in large language models (LLMs) have demonstrated potential to improve AV interface interactivity, potentially filling the social and informational gap left by the driver  [18, 22, 23]. Despite the potential benefits, commercial robotaxi services have not yet integrated such systems. Spatial cognition studies indicate that active engagement, such as utilizing spatial abilities, narrative instructions, or spatially referencing landmarks, can foster *passenger* spatial learning that the established passivity suppresses [11, 13, 14, 19, 21, 24, 25]. This raises a design question of how a conversational user interface can be designed that both facilitates engagement and spatial learning for the passengers.

To address this question, this paper introduces RideGuide, a multimodal, context-aware CUI designed to take on the role as a tour guide and conversational partner in a robotaxi context. Passengers interact with the system via seat-back displays, showing an interactive map and a continuously listening voice interface. The system utilizes the OpenAI *GPT-4o* model to generate contextually relevant and human-like responses, which it outputs through text and an OpenAI’s text-to-speech (TTS) audio stream. RideGuide adapts to preferred interaction language, TTS voice gender, and chatbot personality. To summarize, the paper makes the following contributions:

1. Design and implementation of RideGuide, a customizable multimodal CUI (voice, touch, visual, and spatial data) that serves as a context-aware tour guide for robotaxi passengers. The system uses a runtime intent detection pipeline to filter queries based on chat, directional visual query, landmark name, and category. To enable reproducibility, the source code and datasets are made available (see supplementary information).
2. Findings from an exploratory lab study (*n* = 12) with a pre-recorded ride through a touristic area in Zurich, Switzerland, to understand passengers’ preferred interaction modalities, TTS voice gender, language, and chatbot personality in an AV.
3. Design recommendations for future multimodal robotaxi CUI systems, based on a mixed-method lab study.

While spatial learning is the long-term goal, this study primarily explores how multimodal conversational interfaces can foster passenger engagement with their spatial surroundings in robotaxis, thereby creating the preconditions necessary for spatial learning. Given the lab setting, the emphasis is on qualitative and preliminary quantitative findings (e.g. landmark recall) rather than longitudinal outcomes.

## Related work

### Multimodal interaction with maps

Multimodal interaction lets users combine complementary input modalities, each contributing according to its strengths [26]. Speech is well-suited to expressing temporal aspects or referring to out-of-view objects, whereas pen or touch input is more precise for specifying spatial locations [26–28]. Maps have long been a canonical medium for this kind of pairing, building on a tradition of pen-based input that evolved from paper to digital stylus and touch surfaces [26, 28, 29]. The benefit is most noticeable in spatial tasks, where users adopt multimodal interaction in the large majority of cases, with reported rates of up to 86% [27]. However, realizing such interfaces requires architectures that fuse multiple input streams into coherent actions [28]. These architectures should follow established principles for multimodal design to minimize cognitive load through temporal coordination of modalities (e.g., highlighting a place on the map in sync with verbal output) while ensuring users remain aware of the modalities available to them [27, 30]. In the vehicle, however, these principles have seen little use. In-car comparisons of conversational and touch-based interfaces point to complementary strengths for information access and control [31], but how speech and touch can jointly support a passenger’s spatial learning during a ride remains largely unexplored.

### Passenger engagement in autonomous vehicles

Over the past decade, the development of autonomous vehicles (AVs) has progressed from no automation (Level 0) to full self-driving under certain conditions (Level 4), enabling the growing adoption of robotaxis in major cities around the world [3, 32]. The level of automation is measured on the scale of the Society of Automotive Engineers (SAE), with full self-driving (Level 5) presenting the highest level of automation [32]. As AV capabilities improve and adoption increases, passengers benefit from improved safety by mitigating human error, lower fares compared to traditional taxis, convenience, efficiency, and security, especially for women and elderly people [5–7, 33, 34].

However, with its positive implications, the adoption of AVs also comes with a shift in how passengers engage on their journeys, as the human driver who provided local insights and personal interaction is no longer present [6, 10]. Recent research has indicated that passengers will engage in journey unrelated tasks, such as work, entertainment, or rest  [8]. It creates an isolating environment, where the lack of conversations reportedly negatively influences robotaxi adoption for elderly passengers [35]. When using turn-by-turn navigation apps, users often rely on the app to guide them, which can negatively affect their spatial abilities and memory [14–17, 25]. While navigation apps still require users to follow directions and actively engage with their surroundings, passengers can travel from point A to point B without actively participating in the wayfinding process. In 2010, Mondschein et al. already demonstrated that car passengers build up inaccurate cognitive maps [11]. Based on an online survey (*n* = 204), Qin et al. have laid out early evidence, that frequent AV users have worsened self-reported spatial knowledge [13]. Acquiring spatial knowledge is important, as it facilitates user autonomy, making navigation technology-independent wayfinding and orientation in known environments possible [16, 20, 21, 24]. Effectively engaging passengers in spatial learning depends on attention allocation and cognitive load, as active engagement can redirect attention towards the surroundings, but can also increase cognitive demands that potentially counteract the learning effect [14, 24, 25, 36, 37].

An increased adoption beyond daily commutes, with autonomous tourism expected to change the preferred mode of transport towards to AVs, including long-distance bus and air travel [8, 38], further emphasizes the relevance of this issue. Nevertheless, it also creates an opportunity for future robotaxi interface design that engages passengers in activities previously fulfilled by the missing human driver.

### Conversational user interfaces in autonomous vehicles

Robotaxi passengers engage in passive travel , making them free to engage in any activities, including spatial learning [8, 11]. While there are tools and methods to support spatial learning, effectively engaging passengers ultimately depends on the systems in place to facilitate such interaction and inherent motivation for interaction.

Current commercial robotaxis use in-vehicle displays to convey important information, with services like Waymo One allowing passengers to view sensor data (camera, lidar, sonar) through interactive 3D visualizations that show the vehicle’s surroundings and planned path, alongside contextual explanations for vehicle maneuvers (e.g. yielding to pedestrians) [39]. This limits the scope of possible in-car interactions (i.e. touch input, visual and audio output) and simultaneously impacts accessibility for visually impaired individuals [40]. A recent field study indicated that bidirectional voice-based guidance by a navigator (passenger or CA) can improve the drivers’ landmark and route recall by also referring to environmental details (i.e. landmarks), while adding no extra workload [18]. Speech-based interfaces in cars increase convenience by enabling dialogues between passengers and the vehicle, providing information and control over the in-vehicle environment [41, 42]. In this context, CUIs represent a bridge between static seat-back displays and more accessible and flexible interactions [31, 42].

The premise of this research is to explore an in-car robotaxi interface substituting the missing human driver, passengers inherently want to converse with and to learn from. Currently, ride-hailing apps for traditional taxis allow passengers to select drivers based on factors such as fare, driver rating, and, in some cases, gender [43]. The driver can impact how comfortable a passenger feels and their willingness to engage in a conversation  [6]. In contrast, conversational agents (CAs) can be designed to act context-appropriately, meeting user preferences and expectations to improve engagement and satisfaction [44, 45]. For instance, user-aligned customizing of the voice characteristics, such as the gender, can subtly influence trust and perceived competence [46, 47], understanding and communicating in the user’s mother tongue, dialects, and cultural norms can improve authenticity and accessibility [48] and consistent chatbot personality can make interactions more comfortable and engaging [45, 49]. The use of a self-personality-aware chatbot (SPAC) that has their own defined personality traits can support the adaptation of role-defined agents with user preference and expectation [45, 49]. Therefore, CAs in AVs can be adapted to the passenger, including its interaction language, gender, and personality. Through the advancements in LLMs, modern CAs slowly evolve from providing static information to dynamic and user-adapted conversation partners, that can process open-ended inputs and generate human-like, context-aware replies [22, 45].

### Conversational tour guide

In traditional taxis, passengers can talk to the driver, engage in small talk and request local information [6, 10]. While it is difficult to infer active engagement for all passengers’, the driver thereby acts as a personal tour guide, verbally anchoring landmarks in conversation through spatial orientation and thus assisting passengers spatial learning [18, 24]. Early studies on AV CAs confirm that passengers still expect such a role once the human driver disappears. Through guided brainstorming (*n* = 9) and Wizard-of-Oz rides (*n* = 12), Rege et al. identified tour guide, advisor, and storyteller as potential roles for conversational interfaces in AVs [22]. The tour guide role therefore provides a natural starting point for designing in-car CA that counteracts passive travel. In general, Rege et al. proposed design guidelines for conversational AI (CAI) in AVs, these include: task-independent conversational partner, context-awareness, communicating limits, ethical data usage, not overwhelming or annoying, user-centric personalizations, and implementing a feedback-loop [22]. Furthermore, guidelines for CAs that are capable of local information retrieval were reported by Acer et al. in 2019. Based on a survey (*n* = 1992) and interviews (*n* = 21), participants reported a preference for voice-based interactions, spontaneity, reactive (user-initiated) rather than proactive information delivery, and local information (events, safety, infrastructure, health) [23].

CUIs introduce in-car interactivity, but sustaining spatial learning requires the interface to anchor the conversation in the spatial context of the trip. There have been many approaches to designing interfaces that served a similar purpose by providing contextually relevant information in an interactive interface. Early work by Cheverst et al. in 2000 demonstrated a context-aware handheld system (GUIDE) that used manually curated and contextually relevant information to guide users through the city, with tourists rating it as useful and reassuring [50]. Over the years, manual guides became more dynamic. In 2008, Schöning et al. demonstrated how geotagged Wikipedia data can be utilized to automatically generate narrative audio tours. And by using the phone’s camera and analog maps instead of GPS, it was one of the first multimodal tour guides [51]. More recently, Belz et al. utilized LLMs to dynamically generate audio stories that synchronize in real-time with nearby landmarks in a moving vehicle. Participants showed improved landmark recall when compared to AI-generated stories that were not contextually aware [52]. Finally, conversational in-vehicle navigation instructions have been shown to be effective with significantly higher landmark recognition and more environmental engagement [18], indicating that two-way dialogues can be used to improve spatial knowledge acquisition. Context awareness and spatial orientation are important for supporting spatial learning, with landmarks playing an essential role in developing and improving the cognitive map [19–21]. A future system needs to combine context-aware landmark audio narration with an appropriate tour guide persona to anchor the CA dialogue in the spatial context of the trip.

### Summary

While historical multimodal map interfaces successfully integrated speech and touch interaction [26, 28], they were often constrained by predefined grammar and command vocabularies. Augmenting these established multimodal design principles [27, 30] with the reasoning capabilities of LLMs, conversational agents can now process complex inputs. Consequently, this paper explores the integration of multimodal conversational interfaces into in-vehicle seat-back displays commonly used in commercial robotaxis, aiming to promote active user engagement in spatial learning and to ground a better understanding of user preferences as well as expectations for conversational companions in robotaxis. To design an adequate CUI for the presented role, the conversational agent has to be linguistically accessible (language), aware of the spatial and conversational context, and passengers need to be motivated to interact with it, given individual expectations and preferences.

## RideGuide: design & implementation

Building on prior work, RideGuide was designed as a multimodal conversational interface for robotaxi passengers. The goal was to explore how a familiar robotaxi in-car display could be extended to act as a spatially aware conversational companion, supporting passenger engagement and offering local insights adapted to individual preferences. RideGuide is implemented as a Flutter-based mobile app integrating touch, voice, and map interaction, combined with LLMs using user query, user customization, geospatial and visual data as input to attempt recreating the informative and social interaction provided by human drivers.

### User interface design & interaction

The system is currently divided into an onboarding and a main interaction screen.

**Onboarding:** RideGuide provides an onboarding interface that introduces the system interaction, explains the study task, and allows customizing the system. This study focuses on gathering insights on how participants would freely describe their ideal conversational robotaxi chatbot, i.e., a self-personality-aware chatbot (SPAC) [45], preferred TTS voice gender (feminine = *echo*, masculine = *nova*), and interaction language limited to 60 supported languages by the OpenAI TTS model (*tts-1*) [53].

**Fig. 1:**
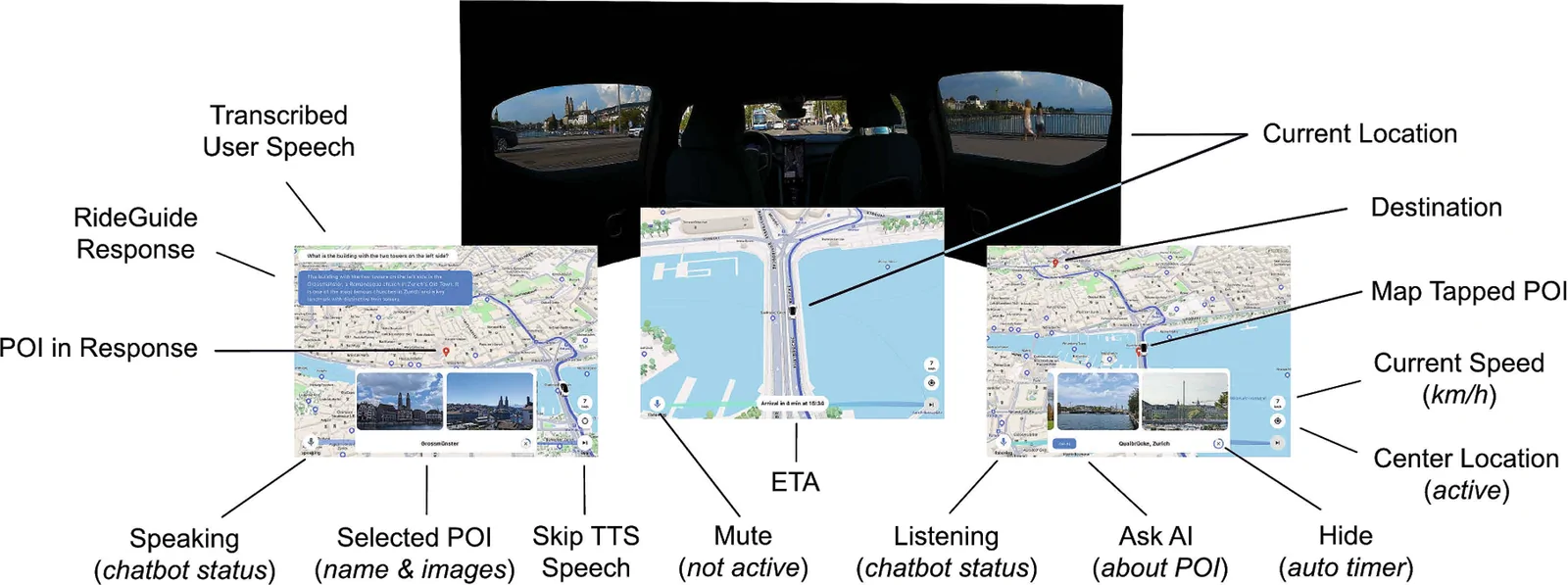
The RideGuide main interface illustrated: (*left*) after a directional voice query referencing a visible point of interest (POI), (*middle*) the default listening, (*right*) the touch interaction state after tapping a map POI, showing the POI image gallery and the *Ask-AI* button that grounds a subsequent voice query in the selected location. The map uses Mapbox with OpenStreetMap and Wikipedia place annotations, as well as Wikipedia images (*in-app images replaced in figure*) [54, 55]. © map data by Mapbox, OpenStreetMap contributors [54, 56]

**Map Interface:** The main interface is illustrated in Fig 1 with the default state in the center, interaction with directional voice queries on the left, and map interaction on the right contextualized by the backseat passenger view of the current position on the top. It uses a 3D map with a custom style using touristic points of interest (POIs) from OpenStreetMap and geotagged Wikipedia articles [54, 55]. It is centered on the current location of the car to support self-localization [16] while the map itself supports the acquisition of spatial knowledge [15, 57].

In this context, *multimodal* refers to the combination of voice (speech input and TTS output) and touch (map gestures and button selection) as complementary input channels, where each modality can be used independently or in sequence to complete a spatial or conversational query. Users can pan and zoom the map freely using standard touch gestures, tap any POI marker to open its image gallery, and tap the *Ask-AI* button that appears upon selection to ground a follow-up voice query in that location. Letting users distribute their input across complementary modalities [26] supports greater accessibility and flexibility [31].

To manage cognitive load, the system supports sequential rather than simultaneous multimodal inputs. The system continuously listens via on-device speech recognition without a wake word, so the user can follow up directly. The transcribed input is updated in real-time in a white text box at the top left. The chatbot responses are typed into a blue output box underneath it and are read aloud using OpenAI TTS synthesizer. While speech-only interfaces can lead to a high cognitive load, visualizing and summarizing the conversational text helps support the interaction, and simplifies follow-up questions [31, 41]. Furthermore, the system supports synchronized multimodal output [30]. When the chatbot references a landmark, whether initiated by a map tap or by a passenger asking about a place they see outside the window, it provides auditory TTS feedback while the map automatically pans toward the referenced landmark and displays associated images [58]. This synchronized multimodal feedback loop supports spatial orientation and knowledge acquisition [19, 21, 24].

Further user control is facilitated by two buttons to mute the microphone and to skip the currently played TTS response. Finally, the interface reflects its current status using text and status colors at the screen bottom (i.e. *listening*, *thinking*, *speaking*, *muted*) and displays the estimated time of arrival (ETA), arrival time, or vehicle speed, loosely mirroring Waymo’s passenger interface [39].

### Chatbot design

RideGuide utilizes *GPT-4o* [53] to generate contextually relevant responses. The system takes into account multiple contextual variables when formulating answers: the current user query and related intent, conversation history, user-defined personality, language, contextual information (i.e. location, heading, visual surroundings) and a local database of relevant POI. By combining these inputs, the chatbot maintains a coherent dialogue flow and provides location-aware responses to the passenger’s questions. This allows the chatbot to handle follow-up questions and to refer to earlier topics or places mentioned.

To understand and merge these sequential multimodal interactions, RideGuide utilizes the LLMs conversation history to achieve contextual fusion [26]. When a user initiates an interaction via the map interface by tapping a POI and selecting *Ask-AI*, the system bypasses the standard intent detectors and automatically defines the input query as: "*What can you tell me about:* [Landmark]", injecting the spatial and semantic metadata of the tapped location directly into the LLMs context window. Consequently, when the user subsequently speaks a follow-up query relying on spatial references (e.g. "*What is the history behind it?*"), the LLM successfully merges this new auditory input with the previously established dialogue context. This sequential fusion allows the system to generate an accurate, location-aware response without requiring the user to explicitly restate the spatial referent.

#### Data sources

The system has local access to OpenStreetMap (OSM) places that have inherited touristic value (e.g. museums, parks, restaurants), extended with geotagged Wikipedia articles [54, 55]. All places are categorized using the Google Places schema [59] to enable efficient proximity search by category (e.g. restaurants nearby). A museum would thereby be classified in the category Culture. In the lab study, a pre-recorded 270 degree view video of a robotaxi ride was used (see section 4.1), which was analyzed frame-by-frame with the OpenAI vision model *GPT-4o* [53] describing the visible surroundings outside the car (i.e. physical environment, dynamic elements, infrastructure). This allows the user to directly refer to surroundings visible through the car windows in their query. When a directional query is requested, the system uses the current location extracted from the pre-recorded route (GPX file) and connects it with the place database by calculating the vehicular GPS bearing with the queried direction.

#### Voice-Input detection & processing

One challenge in a voice-driven system is distinguishing between the user input and irrelevant speech. RideGuide addresses this with a two-step filtering approach. Users can simply start talking to initiate a conversation through the voice input, and the system will generate a response. Since users can start talking without the use of any keywords or manual activation, RideGuide uses two detectors to understand the user’s intent. An *IntentDetector* checks the input for validity by evaluating transcribed snippets with a *GPT-4o-mini* model [53]. Once a valid input is detected, a *ChatIntentDetector* determines the purpose of the interaction based on four main categories:

1. User wants to chat.
2. User asks about places by name.
3. User asks about the surroundings in a specific direction (e.g. left, right).
4. User asks about places in a specific category.

These were derived heuristically from an analysis of common spatial and exploratory queries expected from urban tourists, differentiating between open-ended casual conversation and task-oriented location retrieval, such as querying places by name, category or relative direction [45, 58]. The selection of these specific categories directly supports the observed *in-situ* information needs of urban tourists. Empirical research indicates that tourist navigation heavily influenced by environmental cues encountered *en-route*, with travellers frequently pausing to inspect, and ask questions about places that come into view or otherwise catch their attention [60]. By explicitly parsing intents, RideGuide computationally mirrors the exploratory behaviour that tourists exhibit when exploring novel environments [60], ensuring the LLM filters specifically for relevant local database queries.

**Fig. 2:**
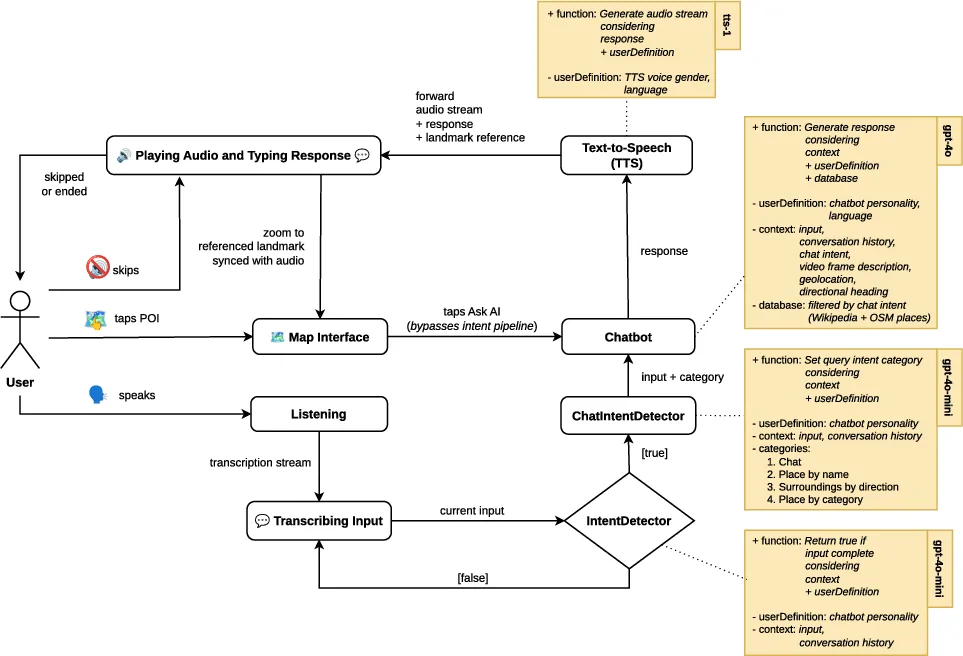
Multimodal interaction routing in RideGuide: Voice input passes through the *IntentDetector* and *ChatIntentDetector* pipeline before reaching the final chatbot output generation. And touch input via map interaction and *Ask-AI* button tapping bypasses both detectors and triggers a spatial query directly. Both paths produce a text-to-speech audio stream and map zoom to the referenced landmark

The final intent detector informs the chatbot about relevant spatial information to filter for the final evaluation request. This improves the response quality, making the outcome more predictable, and decreases the duration needed to generate the response. Conversely, touch-initiated interactions bypass this intent filtering pipeline entirely. When a user establishes a spatial anchor via the map interface (see section 3.2), this is treated as a deterministic intent, so both detectors are skipped. For a simplified illustration of the multimodal routing and the utilized variables and models, refer to Fig 2.

## Methodology

To explore the potential of a multimodal conversational interface in robotaxis, a lab-based pilot study was conducted using a simulated autonomous ride scenario. The goal was to see how RideGuide could foster spatial awareness and engagement, while also capturing user preferences and needs for future system design. The study was framed around the following research questions:

1. How do participants utilize the different input modalities (voice, touch, visual) of the RideGuide CUI when experiencing a pre-recorded ride in a simulated robotaxi lab setting?
2. To what extent do passengers recall landmarks and spatial details after interacting with RideGuide and what interaction patterns facilitate the recall?
3. What are participants’ stated preferences for a robotaxi CUI agent persona in terms of TTS voice gender, language, and personality traits?
4. What capabilities and roles do participants expect of CUIs in robotaxis, and how do these expectations align with or diverge from RideGuide?

For the subsequent analysis of system usage and modality preferences, we distinguish three specific interaction modes. *Conversation* refers to unimodal voice input initiated by speaking directly to the system. *Map-Tap* refers to unimodal touch exploration where the user taps a POI to view images without triggering the conversational agent. Finally, *Ask-AI* represents the sequential multimodal flow in which users shift from open conversation into this mode by tapping a POI and pressing the *Ask-AI* button, which logs the query as a search intent and grounds their subsequent voice inputs in that specific spatial anchor.

### Study setup

**Fig. 3:**
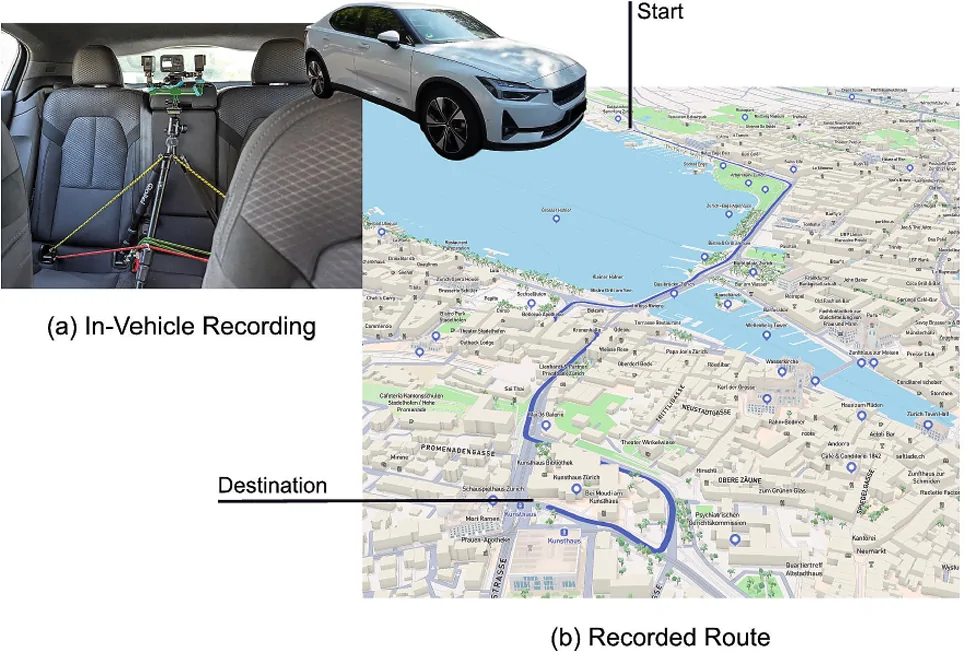
The figure illustrates (a) the GoPro camera setup used to record the ride in the back seat of the car, (b) the pre-recorded route in Zurich, Switzerland using Mapbox with custom OSM and Wikipedia place annotations [54, 55]. © map data by Mapbox, OpenStreetMap contributors [54, 56]

To simulate a realistic robotaxi experience, a 10 min 32 seconds long car ride was recorded using a three-camera GoPro setup with the rear seat (see Fig 3, a). The trip itself was recorded in an electric car (Polestar 2) to allow for capturing road and traffic noises. The video covers approximately a 270-degree field of view, showing the front and sides as seen by a passenger. The selected route was chosen based on visible surroundings (e.g. landmarks) throughout the drive (see Fig 3, b)

**Fig. 4:**
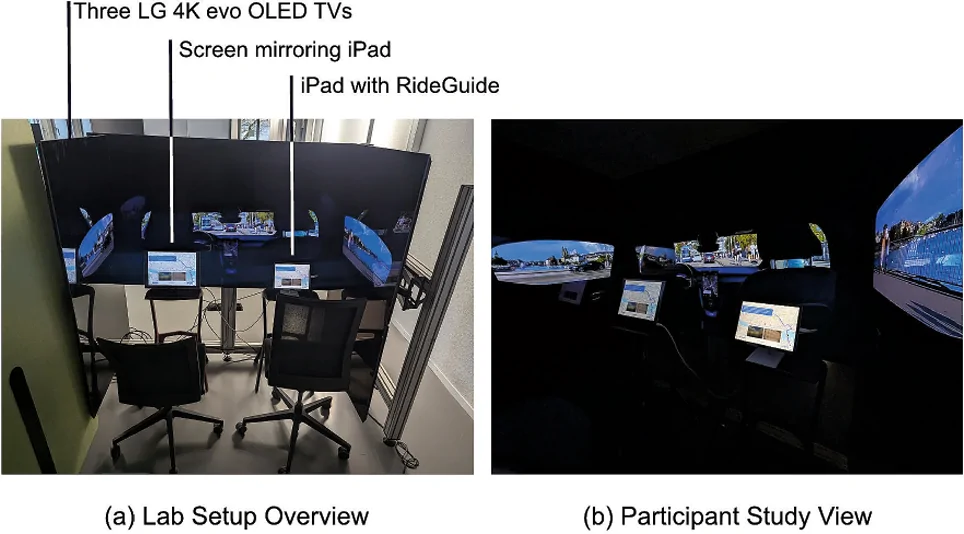
The figure illustrates (a) the setup overview indicating iPad, mirrored screen and TVs, and (b) the participant setup view in the darkened room. © map data by Mapbox, OpenStreetMap contributors [54, 56]

After recording, the videos were stitched together, and the driver was digitally removed from the video footage to create the illusion of a fully autonomous vehicle. The final video was played back across three 65" 4K TV screens arranged to surround the participant, providing a life-size view out of the windows (see Fig 4, a). The environment was darkened, and a soundbar behind the participant played the recorded audio (e.g. road noise). RideGuide ran on an iPad Air (5th gen.) positioned in front of the participant, roughly where a back-seat display would be situated in a real robotaxi. A secondary screen was mounted alongside it to mirror the iPad’s display, mimicking the dual-screen setup of commercial AV systems like Waymo [39]. The setup allowed participants to interact with the system using both voice and touch while experiencing a realistic AV journey (see Fig 4, b).

### Procedure

Each session lasted approximately 45 to 60 minutes. Upon arrival, participants gave informed consent and were briefed on the study procedure. Then, they (1) completed a pre-survey, (2) customized the system by selecting the TTS voice (female or male), choosing the interaction language, along with textually defining a chatbot personality. (3) They were introduced to possible system interactions and the following study task:

*You are visiting Switzerland for the first time and are unfamiliar with the area. You are taking a robotaxi to the Kunsthaus Zurich. During the ride, you plan to learn about nearby landmarks and gain a better understanding of your surroundings.*

(4) Then, participants freely interacted with the system in the pre-recorded ride. (5) Afterwards, participants filled out the post-survey, (6) followed by a semi-structured interview. Finally, (7) participants were debriefed and were able to ask study-and system-related questions. After the study ended, they were given the option to engage with the system again.

### Data collection

The study utilized a mixed-method approach by collecting both quantitative and qualitative data. System interactions were logged on-device, including customizations, map interactions, and conversation data (queries, intents, responses, and duration). The pre-survey data included both custom questions and standardized questionnaires. Participants were asked about their prior experience with robotaxis, conversational habits in taxis, exploration behaviors, familiarity with conversational chatbots, and expectations for a conversational interface in robotaxis. The post-survey assessed perceived ride duration, overall experience, transcription accuracy, response quality, assistance in understanding the spatial surroundings, task performance, and willingness to share personal data in future uses, as well as future conversational likelihood with such a system integrated in robotaxis. Standardized questionnaires included an unweighted NASA-TLX [61], user experience questionnaire (UEQ-S)  [62], and chatbot usability questionnaire (CUQ) [63, 64]. Lastly, collected data included participants’ familiarity with the pre-recorded area and demographic information (age, gender, and mother tongue). For the complete list of questions, refer to Tables 7 and 8.

### Participants

For the pilot study, 12 participants were recruited from the university campus (6 male, 5 female, and 1 undisclosed), with an average age of 26.75, ranging from 20 to 31. Participation was voluntary and uncompensated.

Two participants [P1, P2] were part of a preliminary pilot and were included in the sample. They experienced the same simulation, surveys, interviews and system, though with initial issues of finding places in the database regarding directional system queries (e.g. "*What’s this building on the left?*" [P1]). In the pre-survey, participants indicated moderate intention to use a robotaxi in the future, on a 7-point Likert scale (*M* = 5.08, *MD* = 6.0, *SD* = 1.98). Their motivation for choosing a robotaxi over a traditional ride-hailing service or taxi included novelty (*n* = 7), safety (*n* = 5), solitude (*n* = 5), affordability (*n* = 4), availability (*n* = 3), convenience (*n* = 2), as well as missing language barrier, cleanliness, privacy, and personal entertainment (*n* = 1). Many participants reported frequent use of chatbots or voice assistants in everyday life: 6 very often (6 - 7 times per week), 2 often (4 - 6 times per week), 1 rarely, and 3 never. When it comes to learning about new places, participants reported preferring map apps (*n* = 11), self-guided exploration (*n* = 10), social media (*n* = 10), online review platforms (*n* = 9), and asking locals (*n* = 8). Only 3 participants reported using guided tours and 1 asking taxi drivers for advice. Without prior exposure to RideGuide, participants were asked in the pre-survey about their expectations for a conversational voice-based robotaxi chatbot. Overall, participants anticipated a system that would act as a helpful guide and companion, providing spatially relevant information and local recommendations, proactive and reactive behavior, and interacting with a supportive personality or none (see Table 1).

**Table 1:** Participants’ pre-study expectations for a conversational robotaxi chatbot without prior exposure to RideGuide

Expectation	*n*
Tour guide and navigator role	6
Spatial information	4
Friendly, warm, (emotionally) comforting, engaging, open, informative	4
Local recommendations	3
Reactive and non-intrusive assistant with off-switch	3
Ride details	2
No personality	1
Interesting facts	1
AV technology explanations	1
Human-like conversation	1
Accurate voice detection	1
Higher information accuracy compared to human drivers	1

## Results

This section presents the key findings of the mixed-method lab study, integrating quantitative survey data, system logs, and qualitative interviews. The results are structured along the research questions, focusing on system evaluation, interaction patterns, task performance (spatial recall), customization preferences, and expectations for conversational robotaxi systems.

### User experience, usability & cognitive workload

Participants reported a moderately low workload on the unweighted NASA-TLX using a scale of 1-10, measuring on average 32.4 % (*MD* = 32.5 %, *SD* = 0.45 %). UEQ-S indicated mixed user experiences (0.92), with pragmatic quality below average (0.73) and hedonic quality above average (1.10). The CUQ measured an average score of 67.58 % (*MD* = 73.44 %, *SD* = 21.11 %, *min* = 17.2 %, *max* = 92.2 %) and with moderate transcription accuracy on a 7-point Likert scale (*M* = 3.75, *MD* = 4.0, *SD* = 1.71). Higher transcription accuracy correlated with lower overall workload (NASA-TLX, *r* = -0.72, *p* = 0.008) and higher chatbot usability (CUQ, *r* = 0.82, *p* = 0.001). Overall system experience scored moderately positive on a 7-point Likert scale (*M* = 4.67, *MD* = 5.0, *SD* = 0.99).

### Interaction patterns & modality use

Participants engaged with both the chatbot and the interactive map interface. On average, voice queries were initiated 15.08 times (*MD* = 14.50, *SD* = 4.81). 21.14 % of queries were not further evaluated (*M* = 3.08, *MD* = 2.00, *SD* = 2.50) due to misinterpretations (54.55 %), queries being statements (43.43 %, e.g. "*Oh, OK. I see.*" [P8]) and faulty transcriptions (2.02 %) by the intent detector (refer to 3.2.2). All successful queries were identified as:

- 61.9 % chat

**Figure:**
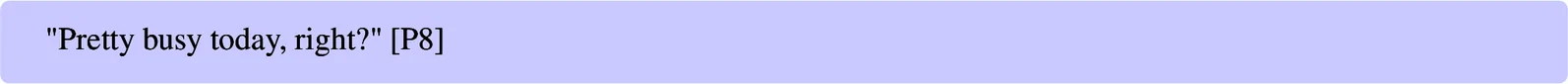
- 14.20 % name

**Figure:**
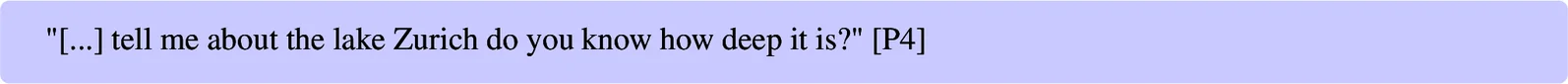
- 13.50 % landmark

**Figure:**
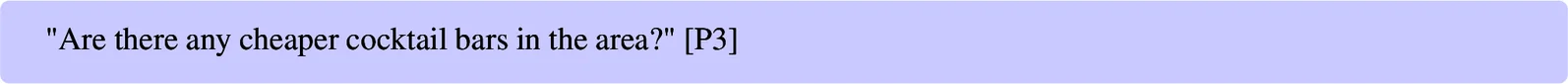
- 10.30 % directional

**Figure:**
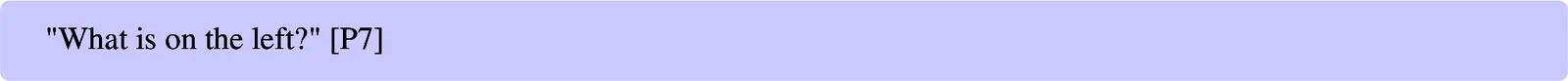

Landmark queries were categorized by the *ChatIntentDetector* as Food and Drink, Entertainment and Recreation, Transportation, Culture, Automotive and Natural Features (see Table 2).

**Table 2:** Categorization of successful queries by the *ChatIntentDetector* including sub-categorization of landmark queries using Google Place categories [59]

Query Category	*n*
Chat	96
Name	22
Landmark	21
Food and Drink	8
Entertainment and Recreation	7
Transportation	2
Culture	2
Automotive	1
Natural Features	1
Directional	16

**Table 3:** Participants’ ratings related to spatial learning and perception of the RideGuide system on a 7-point Likert scale from 1 (Strongly Disagree) to 7 (Strongly Disagree)

Measurement	M (SD)	MD
Prior familiarity with the area	4.50 (2.28)	5.0
Improved understanding about landmarks and route	5.67 (1.30)	6.0
Chatbot information accuracy	5.33 (1.16)	6.0
Chatbot helpfulness for learning about surroundings	5.67 (1.16)	6.0
Map helpfulness for interpreting chatbot responses	6.25 (1.29)	7.0
Voice interaction helpfulness for learning about surroundings	5.83 (1.70)	7.0

The average chatbot response time over all queries was 3.62 seconds per query (*MD* = 3.0, *SD* = 2.79, *min* = 1, *max* = 25), with occasional delays negatively affecting the user experience. Participants described a trade-off between the waiting for replies by the chatbot against the quickly accessible map information.

### Touch screen and multimodal interaction

Five participants actively interacted with the map, averaging 5.4 landmark taps (*MD* = 6.0, *SD* = 2.41) opening its image overview and a further 3.2 *Ask-AI* button taps (*MD* = 2.0, *SD* = 1.64). The *Ask-AI* button produced 14 spatially-grounded queries. Of the 27 map taps, 12 were immediately followed by an *Ask-AI* query, while the remaining 15 were standalone map exploration. These touch interactions complement the place name queries reported in section 5.2. (Table 2), whereby out of the 22 name queries, 14 were issued through the *Ask-AI* button and 8 were spoken place queries, showing that the spatial anchor (see section 3.2) could be established either by touch or by speech. The *Ask-AI* button was used for simple queries, and voice interaction for open-ended follow-up questions:

**Figure:**
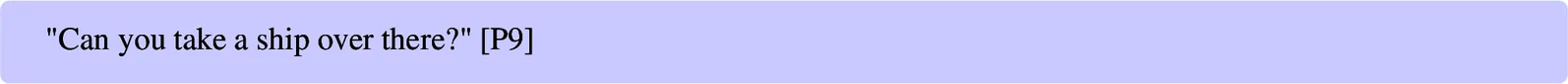

**Figure:**
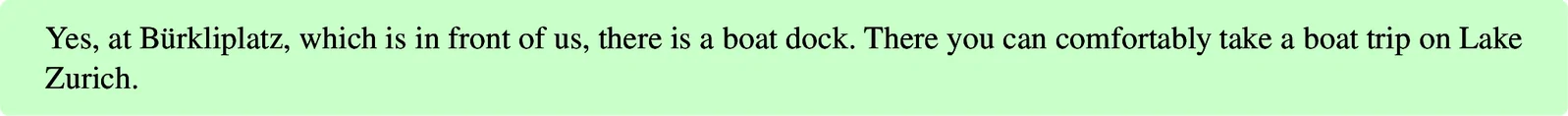

**Figure:**
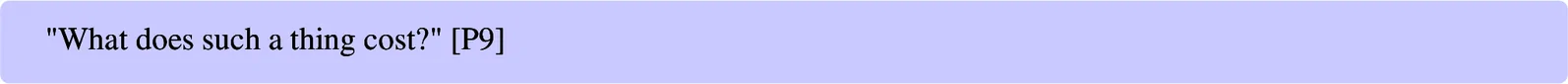

**Figure:**
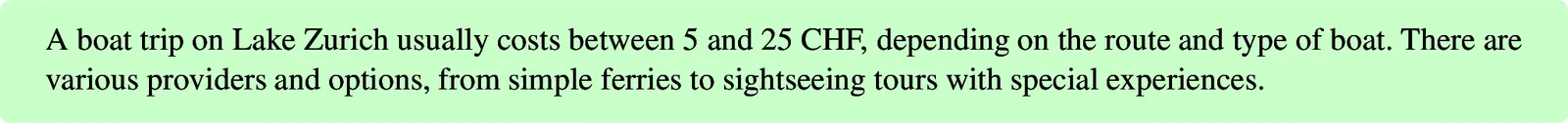

P5 noted that the map "*helped a lot*", but still used the chatbot for deeper information retrieval, as P8 emphasized considering its ability to offer "*insider information*" or recommendations, as the exemplary query by P4 shows:

**Figure:**
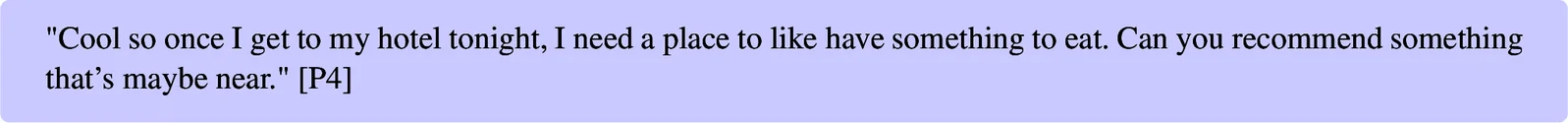

Others found it more natural to ask the chatbot directly rather than searching the map, citing ease of use: "*I just asked what was around instead of looking at the map, it was easier.*" [P7]. Further, the chatbot was used to access detailed contextual information:

**Figure:**
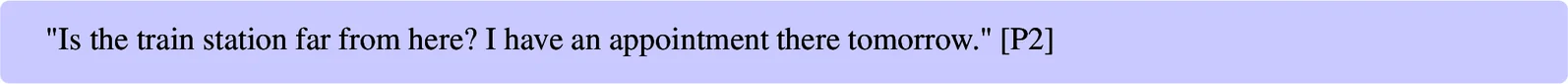

**Figure:**
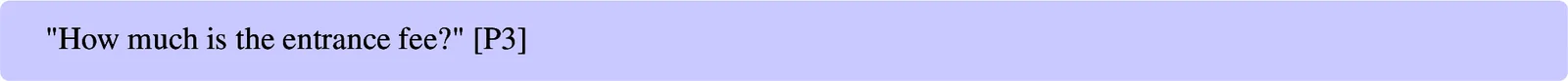

**Figure:**
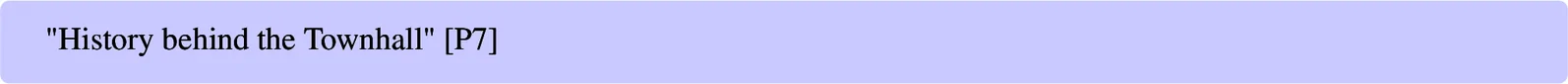

**Figure:**
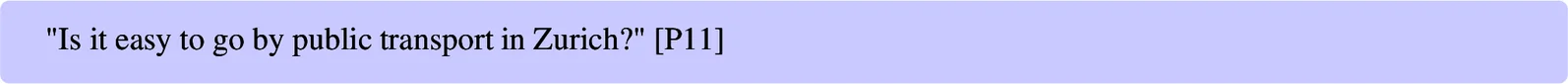

Active voice interaction was limited due to personal preferences and system limitations, but sometimes favored for its narrative quality. In follow-up debriefings, participants became more open to switching between voice and map interactions, suggesting repeated exposure potentially shifts the modality preference.

### Task performance & spatial recall

RideGuide was designed for passengers in a robotaxi to engage in conversations that support spatial learning, predominantly to acquire landmark knowledge. Participants described the simulation environment as authentic, reporting that they perceived it as a real robotaxi ride. Prior familiarity with the areas was reported as moderate, and participants felt more informed about nearby landmarks and gained a deeper understanding of the route through the system interaction. The information the chatbot provided was perceived as accurate and helpful for learning about the surroundings, as was the voice interaction. The map interface was rated as helpful for interpreting the chatbot’s responses (see Table 3).

The ride duration (10 min 32 seconds) was slightly underestimated with an average of 9 min and 59 seconds (*MD* = 10.0, *SD* = 3.22). In follow-up interviews, participants recalled on average 3.33 landmark names they discussed with the chatbot (*min* = 1, *max* = 5), while additionally describing on average 1.42 places based on their purpose or location. Considering that the ride was recorded in Zurich, Switzerland with (Swiss) German place-names, native speakers were the ones that recalled slightly fewer landmark names compared to non-native speakers. Refer to Table 4 for an overview of landmark recall results.

Conversations were the dominant interaction providing landmark information, but high counts of discussed landmarks did not automatically lead to better recall.

**Fig. 5:**
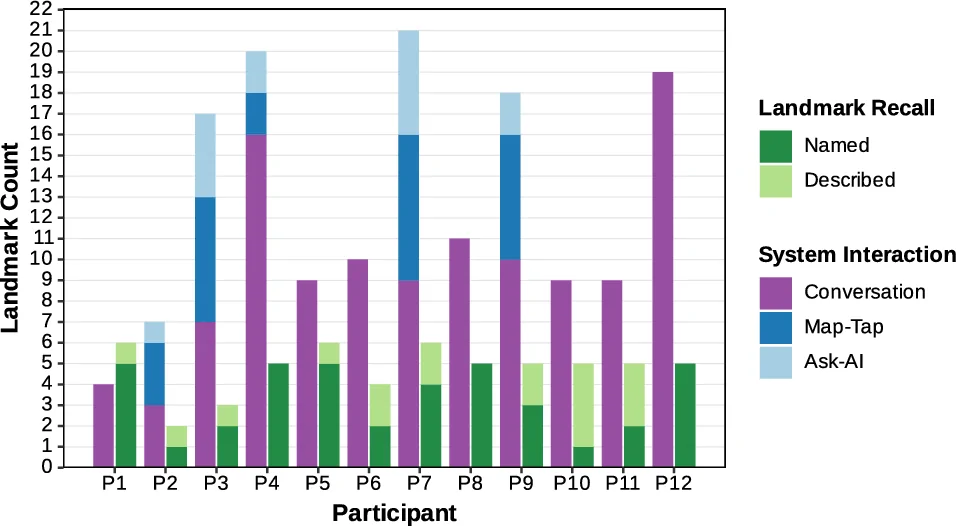
Per participants breakdown of the three interaction modes (see section 4) and subsequent landmark recall. *Conversation* counts voice turns recorded in the chat log, *Map-Tap* counts unimodal POI taps without invoking the chatbot and *Ask-AI* counts touch-grounded queries. Recall shows the number of landmarks each participant later named by label or described by purpose in the post-ride interview

Participants that interacted with the map (*n* = 5) recalled slightly fewer landmarks (*M* = 3.00 vs. 3.43). Fig 5 highlights the comparison between dominant interaction type and the number of landmarks recalled per participant.

During the interviews, participants reported to being stuck looking at the map [P3, P7], with 3 participants actively noting cross-referencing map viewed POIs through the windows [P2, P4, P9]. An interesting example was given by P4, who reported learning about a bridge they saw through the car window: "*Then I thought it was really cool, the Quai Bridge, so to speak, the bridge that you drove over, and I’ve now also memorized the Münster Bridge, because you could see it out of the window on the left, and then I asked about it and it stayed in my head.*" [P4].

### Customization & Preferences

Participants were able to customize RideGuide during the onboarding process, by selecting TTS voice gender, language, and defining a chatbot personality.

#### TTS voice gender & language preferences

The TTS voice selection was valued, with 7 participants choosing the feminine voice (male = 5, female = 2), and 5 for the masculine voice (female = 3, male = 1, gender undisclosed = 1). Participants reported the language choice as a meaningful decision, beneficial for travelling to improve accessibility, but also to create a feeling of connection when choosing the mother tongue. It was also noted that choosing the local language could help to preserve the cultural integrity when travelling. Four participants interacted with the system in their mother tongue, and 7 opted for English, mentioning expectations of better performance or lack of native language support (see Table 5). Non-native language selection among participants (*n* = 8) resulted in a significantly lower speech recognition accuracy (*r* = 0.65, *p* = 0.02) on a 7-point Likert scale (*M* = 3.00, *SD* = 1.51) compared to native language selection (*n* = 4, *M* = 5.25, *SD* = 0.96) with accent discrepancies worsening the transcription accuracy.

**Table 4:** Participants’ landmark recall rate based on the name, compared by native and non-native speakers and description of purpose. (Swiss) German speakers are considered native to the place names in the lab study

Landmark Recall	M (SD)	MD
Name	3.33 (1.67)	3.5
Native speakers (n = 7)	2.71 (1.70)	2.0
Non-native speakers (n = 5)	4.20 (1.30)	5.0
Description	1.42 (1.24)	1.0

**Table 5:** Language selection based on participants’ native (mother) tongue

	Selected		
**Native**	Chinese	German	English
German		3	3
Swiss-German$^{1}$			1
Chinese	1		
French			1
Bosnian		1	
Malayalam$^{1}$			1
Filipino			1

$^{1}$ Language was not available in the system

#### Chatbot personality customization

Following the pre-survey, participants defined their own chatbot personality via a free text input during onboarding of RideGuide, described by the following coded themes: humorous, informative, professional, friendly, playful, user-controlled, and storytelling-oriented. Interaction style preference can be divided into reactive behavior (*n* = 7, i.e. responses to direct queries) or proactive behavior (*n* = 5, i.e. dynamically initiating conversation). However, proactive behavior was generally expected to remain limited to contextual relevant information (e.g. nearby POIs).

In the post-study survey, participants reported moderate alignment between the chatbot behavior and their described chatbot personality during onboarding (*M* = 5.08, *MD* = 5.0, *SD* = 1.51). For 3 participants, it exceeded expectations (e.g. knowledge base, engagement), 3 reported their expectations were met due to its engaging style and lack of pressure. Another 3 had mixed impressions due to issues such as incorrect input handling, missing reactivity and multimedia control. And 2 reported having no clear expectations, and one participant was disappointed. Retrospectively, participants expressed their interest in the chatbot being more enthusiastic, witty, charming, fun, or local. Others appreciated the friendly and informative tone or preferred a bland or neutral style. During the interviews, participants emphasized a preference for dynamic chatbot adaptations over static definitions, suggesting the use of predefined personas, evolving personalities based on user preferences, as well as options to edit the personality during the robotaxi waiting period in future real-world implementations.

### User expectations for conversational robotaxi systems

The following sections expand on these expectations altered through exposure to RideGuide, and present participant’s expectations for a similar system in future robotaxis based on the surveys and interviews.

#### Functionalities & knowledge capabilities

RideGuide met certain functionality expectations, but also revealed limitations and areas of improvement that participants described in the interviews. The current system was unable to answer questions about dynamic or temporary events, limiting its usefulness (e.g., crowds, content on posters, pedestrian’s clothing):

**Figure:**
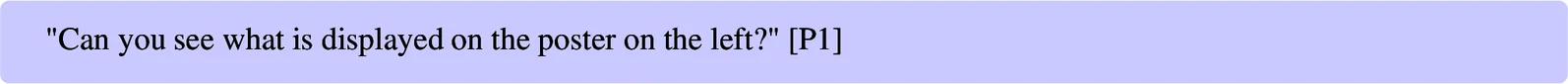

**Figure:**
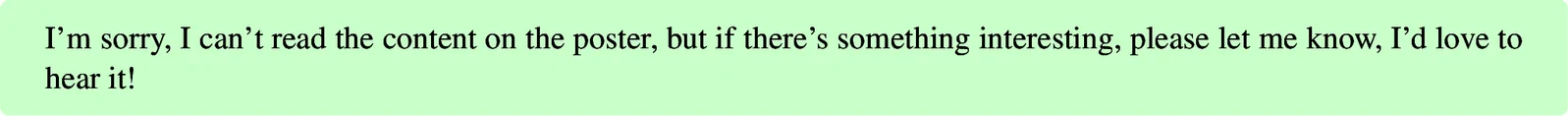

Real-time information about traffic, ticket pricing, navigational directions, events, opening hours, venue seating availability, and public transport schedules was frequently referred to as being essential. Some attempted to ask about objects or situations outside the predefined knowledge base, indicating an expectation for a broader and more flexible knowledge base.

Several participants wanted RideGuide to proactively engage based on current surroundings, such as noticing and commenting on visible events or landmarks, but also stressed that this proactivity should be context-aware, time-limited, and user-controllable. In addition, participants expressed interest in discussing current local events and engaging in small talk, while others preferred to keep the conversation focused on practical or spatial information. For instance, one participant remarked: "*I didn’t know that Karl Cox once played at Street parade. I thought that was really cool. And also that Giacometti is represented in the Kunsthaus.*" [P8].

#### Likelihood for conversational engagement over taxi drivers

Participants reported their likelihood of engaging in conversations with a taxi driver (pre-survey) and with a voice-based robotaxi chatbot (post-survey), on a 7-point Likert scale (see Table 6). In familiar environments, participants showed overall reluctance to engage in conversations with both taxi and a robotaxi chatbot, indicating a preference for limited conversation. In unfamiliar environments, conversational willingness increased, reaching neutral to positive ratings, with a stronger preference for interacting with the robotaxi chatbot.

**Table 6:** Participants’ conversational likelihood with taxi drivers (pre-survey) and RideGuide-like voice-based robotaxi chatbot (post-survey) on a 7-point Likert scale ranging from 1 (Strongly Disagree) to 7 (Strongly Agree)

	Familiar		Unfamiliar	
	M (SD)	MD	M (SD)	MD
Taxi driver	3.25 (1.22)	3.0	4.42 (1.51)	5.0
RideGuide	3.33 (2.10)	2.5	5.42 (2.15)	6.0

Three participants

positively remarked the ability to remain silent, noting similarly feeling more comfortable with a silent robot than a quiet human, since in the end "*the robot doesn’t care*" [P5]. Nine participants indicated they would even converse with RideGuide when accompanied by a friend or family member, especially to learn together, test system limitations, or include the system in the group.

#### Conversational preferences

The pre-survey assessed participants’ conversational preferences when interacting with conversational chatbots, using a 7-point Likert scale ranging from 1 (strongly information focused) to 7 (strongly conversation focused). In the pre-survey, participants reported a preference for information-focussed conversations with context-independent conversational chatbots (*M* = 2.83, *MD* = 2.5, *SD* = 1.27). After experiencing the RideGuide system, the question was repeated in the post-survey, specifically regarding their future conversational preference for a RideGuide-like robotaxi system. Here, preferences were directed towards a more conversation-focused style (*M* = 3.75, *MD* = 4.0, *SD* = 1.77), indicating increased openness towards engaging in more casual or exploratory dialogue. There are good examples in the interaction with RideGuide (see Supplementary Materials), for instance:

**Figure:**
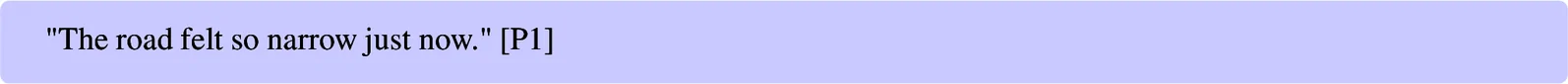

**Figure:**
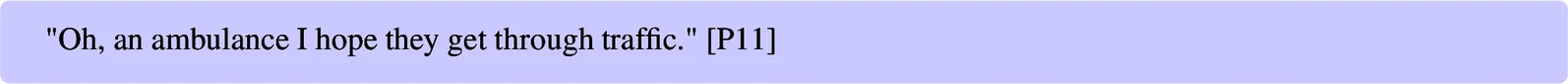

During the interviews, participants outlined topics they would like to discuss with RideGuide in future interactions, which included recommendations about places, events, or activities, spatial questions such as map content, specific locations, or real-time information, and practical information like ticket pricing or weather. These are detailed in the previous section.

Additionally, they expressed interest in more casual, companion-like interaction. Suggestions included discussing the surroundings , having the system suggest activities based on the current location, weather and the view (e.g., going to the beach [P6]), or treating the chatbot as a conversational partner. Proposed topics ranged from getting to know each other [P12], sharing funny anecdotes [P3], writing poems [P1], or discussing cultural aspects [P2], sports and politics [P8], to seeking personal advice [P11], hearing about the chatbot’s lived experiences [P4], or having it complain about traffic [P6]. These preferences reflect potential directions for future iterations of RideGuide. However, not all were interested in extended conversation, e.g. P10 noted they would prefer to walk rather than engage in such dialogue, similar to their reluctance to converse with a taxi driver: "*And if there’s an awkward silence in the car... I don’t like taking a cab at all, [...] then you get in, then there’s a stranger, then you’re somehow forced to talk to them, even though you don’t feel like it. So I find that very annoying and to be honest, I’d rather walk than have forced conversations with someone because I think to myself [...]. So I think that’s one advantage of it, that you can just really ignore all these human sensitivities and do what the user wants to do at that moment.*" [P10].

#### Data-Sharing

When it comes to data sharing with RideGuide, participants had mixed feelings. Specifically, personal questions from the chatbot could be seen as intrusive: "*Why are you traveling here? [...] I would be suspicious that the robot is trying to steal my personal data.*" [P5]. However, the given participant simultaneously acted contradictory as they elaborated on their dating experience with the system during the study:

**Figure:**
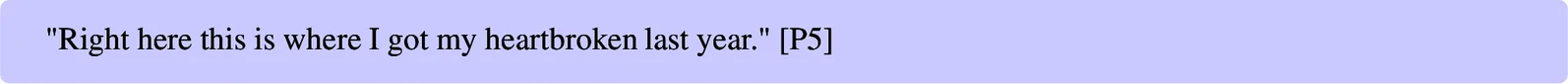

**Figure:**
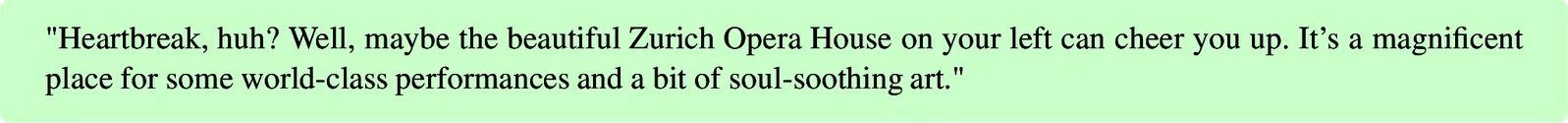

On the other hand, some participants reported openness towards personal questions, expecting a sense of companionship: "*Like more like getting to know each other. [...] What’s your name? Where have you been? [...]*" [P12].

According to the post-study survey, 66.67 % expressed willingness to share personal data to enhance the chatbot’s personalization. This included conversation preferences (e.g. topics, communication style) (*n* = 8), navigational preferences (e.g. preferred route) (*n* = 7), location history (e.g. past destinations, landmark interests, familiar locations) (*n* = 5) and conversation transcripts (*n* = 4). Participants emphasized that a chatbot perceived as having local knowledge or personal experiences could foster deeper engagement, as P4 explained: "*If I knew that there was a person sitting there who had lived in Zurich for 30 years, so to speak, and knew all the little corners and bars and things like that, then I think I would ask different things than I would have asked a system like that now. So, ask even more about lived experiences.*".

During the interview, P4 further suggested the idea that RideGuide could crowdsource local stories and knowledge from passenger conversations to provide more local insights for future users. Building on this, 6 out of 9 participants expressed willingness to share their conversations as anonymized stories.

## Discussion

The aim of this study was to explore how a customizable, multimodal conversational interface can foster engagement and spatial learning for robotaxi passengers to compensate for the absence of a human driver. Essentially, the goal of this work was to recreate some of the informative and social value a human driver can provide. While the primary focus was on active engagement, the findings provide preliminary evidence that such interfaces can support spatial knowledge acquisition to counteract passive travel as a risk in autonomous mobility [13]. This section discusses how RideGuide facilitated spatial engagement and learning, and identifies design implications for future in-vehicle CAs based on reported preferences and expectations.

### Facilitating spatial learning in robotaxis

The results indicate that RideGuide fostered active engagement with the environment, with participants recalling on average 3.3 landmarks by name after the ride and rating the chatbot as helpful for learning about the surroundings. Participants slightly underestimated the ride’s duration, implying an engaging experience, which coincides with prior studies linking engagement to improved spatial memory [14, 25]. The map interface was used for immediate information acquisition and the voice interface for follow-up questions. Similar to Antrobus et al., the information quality of the "navigator" was perceived as accurate (see Table 3) [18]. Actively asking questions, listening to contextual information, and cross-referencing the location of discussed POIs on the map with the visible surroundings outside the car helped foster active spatial learning. The coexistence of both a map and voice interface made information more readily accessible, while also supporting the preference for reserved interaction through the sole use of the map interface. Some participants commented that simply asking RideGuide about places they saw through the window fueled their interest in learning actively about their surroundings, since it did not require effort for manual research (see section 5.4). Interestingly, given the importance of balancing engagement and cognitive load for spatial learning [12, 14, 24, 25, 36, 37] and active engagement increasing cognitive load [14], participants’ workload remained low, implying RideGuide ’s interactions did not overwhelm them. However, it is important to note that the study was exploratory and lacked a control group to quantitatively measure improvements in spatial learning. Future work should compare the system against a passive infotainment system as a baseline, ideally in a real-world driving context.

### Implications for conversational user interface design

Participants’ stated preferences (both before and after using the prototype) align with prior findings that robotaxi passengers would value a tour guide CA capable of conversational interaction [22]. This section identifies key design considerations for future CUIs in robotaxis, that emerged from the expectations and preferences reported in the study.

#### User-Adapted conversational interface

Personalizations emerged as a critical factor for both engagement and usability throughout the study. Without prior exposure to RideGuide, participants reported interest in the pre-survey for a customizable system (i.e. types of information, chatbot personality and proactive behavior). Participants generally reported that the gender choice was not important, with no measurable impact on the user experience or their perception of the chatbot. While voice assistants often default to feminine voices [46], 58 % of participants chose the same within the study. In contrast, language was the customization perceived as most impactful, as it enabled passengers to interact with the "driver" in their mother tongue regardless of location, while simultaneously supporting authenticity with local languages in foreign contexts. This highlights the benefits of a CA over human as the conversational partner in a taxi, when considering cultural adaptivity [48]. RideGuide was designed to function reactively to assess inherent spatial learning without active prompting, and to limit the pressure of having to speak to the system. However, the results highlight the need for more user-adaptive behavior, since the reactive approach did not meet the expectations of all participants. The desired chatbot interaction behaviors can be classified into reactive behavior in familiar areas and proactive behavior in unfamiliar areas to highlight nearby POIs. Nevertheless, there was individual variation in these preferences, suggesting that the ideal level of proactivity may depend on personal communication style, and different personalities (extroverted vs. introverted) [49]. To inform future uses, 67 % of participants were open to sharing personal data (i.e. conversation preferences, navigation history, chat transcripts) to enable more personalized interactions. However, some expressed privacy concerns in the interview, with one simultaneously sharing personal data by talking to RideGuide in the study (see section 5.6.4). This paradox is not uncommon for interactions with chatbots and points to the need for transparent and ethical data handling practices in future systems [65]. A conversational tour guide should clearly communicate what data it collects and how it is used to maintain trust and confidentiality.

#### Human-Agent-Vehicle collaboration

The study extends prior findings on AV explainability and user control [22], emphasizing that passengers expect CAs not only to provide local insights but also to mediate between themselves and the vehicle. Participants wanted the CA to be aware of the vehicle’s actions and status, thus transparent explainability for driving decisions. For example, when the vehicle slows down or stops, participants expect the system to explain the reasoning, coinciding with related research on CA capabilities in AVs [66]. Participants also expressed expectations for control over the vehicle via the interface, such as opening windows, media control, or requesting route changes [22]. These insights emphasize that future AV CUI cannot solely act as a tour guide but also need to function as a communicator between the AV and the passenger. While RideGuide operated in this study on a fixed pre-recorded route using pre-evaluated visual data, participants expressed a clear expectation for real-time vision capabilities. For instance, to reason and provide information about events (e.g. "*Why is there a crowd?*" [P1]). This implies that a future system should integrate live sensor data from the vehicle as a chatbot data source to enable real-time contextual reasoning. Further extending this idea, participants expressed interest in the chatbot providing live updates on traffic, nearby events, or practical information like ticket pricing and public transport schedules. Expectations for contextual information showed overlaps with requirements reported by Acer et al. for hyper local CA, similarly do the methods used by participants for learning about new places (see section 4.4) [23]. In summary, a robotaxi CUI should utilize vehicular and environmental data in real-time to respond to queries and concerns [22].

#### From tour guide to conversational companion

Many participants engaged with RideGuide not only to query spatial information but also as a conversational partner. Social norms typically directed towards humans, such as expressing politeness and apologies, were observed with 3 participants applying human social conventions to the CA. When comparing the experience to riding with a human driver, some noted that conversations with human drivers can be unpredictable and uncomfortable, as also reported in related work [6], and that the lack of any obligation to converse in a robotaxi was positively reflected upon. They appreciated that the system would respond if they started talking, but did not unwillingly impose on the user. The self-reported likelihood of conversation also reflected a situation-dependent usage, participants in this sample would avoid non-essential conversation in areas they are familiar with (with both taxi drivers and RideGuide), but were much more willing to engage in unfamiliar areas, especially with RideGuide, given it was perceived as optional and nonjudgmental. Participants expressed interest in talking about a wide range of topics, including local gems, personal experiences, local culture, events, and especially stories that are based on experiences. This suggests the system can be treated more as a social companion, substituting the experience with a human driver. However, participants critically noted its capabilities of substituting authentic lived experiences. They further reflected on a RideGuide-like system to source collective local knowledge by sharing anonymized conversations to inform community-informed recommendations and stories. They noted that this could further improve data quality, as crowdsourced information can infer historic names for landmarks. It aligns with the idea of location-based storytelling, but instead of using AI-generated content, participants listen to stories based on authentic lived experiences.

### Summary

While RideGuide ’s current small talk capabilities were limited, the collected data on user expectations can guide future iterations to foster more engaging conversational interactions. Social anxiety and perceived safety play an important role in positively impacting robotaxi adoption [33]. This can be one of the reasons why 67 % reported that RideGuide conversational interface would incline them to choose robotaxis over traditional taxis, particularly for reasons of comfort, perceived safety, or introvert-friendliness. A CA could become an integral part of the vehicle’s interface, tightening the gap between the passenger and the machine. These findings suggest that a well-designed conversational companion for AVs that addresses key social and experiential needs may further contribute to an increased adoption of robotaxis. However, it raises interdisciplinary questions, from its technical feasibility to user psychology, with ethical considerations on long-term trust and companionship. After all, these results have broader implications beyond the robotaxi domain, potentially enhancing spatial awareness across other mobility contexts (i.e. driving, cycling, or walking).

## Limitations & Future work

This study was exploratory with a limited sample (*n* = 12) of predominantly young adults and was conducted in a simulated lab setting. As such, generalizability to real-world robotaxi usage is uncertain, and observed engagement or spatial learning effects may differ in practice. The prototype had some technical limitations, with occasional speech recognition errors, processing delays, and limited conversational flexibility, which impacted the user experience, particularly for non-native speakers. Some participants found the static, one-time chatbot personality setup overwhelming or too restrictive, indicating a need for predefined personas and dynamic adaptive personalization.

Future research should evaluate RideGuide in real-world and longitudinal field settings, incorporate diverse participant samples, and include a control or baseline condition to better assess spatial learning outcomes. Further, considering its conversational companion capabilities, future versions could be assessed through the lens of a partner model (e.g., trust formation and substitution of the human driver). System improvements should focus on speech recognition, real-time context awareness, conversational flexibility, and adaptive personas. Extending multimodal interaction beyond seat-back displays, such as integrating AR-enhanced windows, as suggested in prior work by Kim et al. [67] as well as integration of gaze and point detection, could further support situated spatial learning and engagement for robotaxi passengers.

**Table 7:** Pre-survey questionnaire structured in 5 pages asked before the simulated ride

Question	Answer
*In this study, you’ll briefly experience a ride in a Robotaxi, a car that drives itself without a human driver.*	
**Q1: I would use a Robotaxi in the future if I have access to it.**	7-point-Likert$^{1}$
*Please indicate what best matches how you feel about the following statements.*	
**Q2: What would motivate you to use a Robotaxi over a traditional taxi or ride-hailing service (e.g. Uber, Lift, Didi)?**	Text
*Use keywords that come to mind.*	
*Please complete this questionnaire by reading each statement carefully and indicate what best matches how you feel about the statement.*	
**Q3: I would engage in a conversation with the** ***taxi driver*** **when I am in a** ***familiar*** **environment.**	7-point-Likert$^{1}$
**Q4: I would engage in a conversation with the** ***taxi driver*** **when I am in an** ***unfamiliar*** **environment.**	7-point-Likert$^{1}$
**Q5: Which methods do you use to explore and learn about new places?**	Multiple Choice (randomized)
*Select all that apply.*	
— I explore independently	
— I consult map apps (e.g. Google Maps, Apple Maps)	
— I consult online review platforms (e.g. TripAdvisor, Yelp)	
— I consult social media platforms (e.g. Instagram, TikTok)	
— I use guided tours (e.g. human guides, audio tours, tour-guide apps)	
— I ask locals for recommendations	
— I ask taxi drivers for recommendations	
**Q6: I feel comfortable navigating new environments on my own.**	7-point-Likert$^{1}$
*Please indicate what best matches how you feel about the statement.*	
**Q7: How often do you engage in conversations with (voice-based) conversational chatbots in a typical week (e.g. ChatGPT, Gemini, Siri, PerplexityAI etc.)?**	7-point-Likert$^{2}$
**Q8: How would you characterize the focus of your interactions with these chatbots?**	7-point-Likert$^{3}$
*In this study, you’ll experience a short ride in a Robotaxi. You will have the option to talk to a voice-based conversational chatbot.*	
**Q9: What do you expect from a voice-based conversational chatbot in a Robotaxi setting?**	Text

$^{a}$ 1) Strongly Disagree to 7) Strongly Agree

$^{b}$ 1) Never, 2) Rarely (< 1 time/week), 3) Occasionally (1-2 times/week), 4) Sometimes (2-3 times/week), 5) Often (4-6 times/week), 6) Very Often (6-7 times/week), 7) Multiple Times a Day

$^{c}$ 1) Strongly Information-Focused to 7) Strongly Conversation-Focused

## Conclusion

This research introduced RideGuide, a multimodal conversational interface designed to re-engage robotaxi passengers with their spatial environment. Through a mixed-methods lab study, findings showed that voice and map-based interactions fostered active engagement and enabled landmark recall, suggesting potential benefits for spatial learning in autonomous travel. However, future work is needed to validate real-world engagement, and long-term adoption. The results provide actionable insights for the design of adaptive and human-centered interfaces in the future of autonomous mobility.

## Supplementary information

The Supplementary information includes the participant sample data set and their highlighted chatbot transcripts (translated into English). The code for the RideGuide Flutter app, including its prompts, is publicly available [68].

## Appendix A Questionnaires

See Table 7

See Table 8

**Table 8:** Post-survey questionnaire structured in 11 pages asked after the simulated ride

Question	Answer
**Q1: How long do you think the Robotaxi ride lasted?**	Number
*Please enter your estimation in minutes.*	
**Q2: How would you rate your overall experience with the system?**	7-point-Likert$^{2}$
*Please indicate what best matches how you feel about the statement. The TV/Video setup should not be considered in this rating.*	
**Q3-Q8: NASA Task Load Index (NASA TLX)** [61] **(6-items)**	10-point-Likert
**Q9-Q16: User Experience (UEQ-S)** [62] **(8-items)**	7-point-Likert
**Q17: The system always accurately transcribed my spoken inputs.**	7-point-Likert$^{1}$
*Please indicate what best matches how you feel about the statement.*	
**Q18-Q33: Chatbot Usability Questionnaire (CUQ)** [63] **(16-items)**	5-point-Likert
*Please complete this questionnaire by reading each statement carefully and indicate what best matches how you feel about the statement.*	
**Q34: The chatbot provided accurate information.**	7-Point-Likert$^{1}$
**Q35: The chatbot was helpful for learning about my surroundings.**	7-point-Likert$^{1}$
**Q36: The map interface helped me interpret the chatbot’s responses.**	7-point-Likert$^{1}$
**Q37: Speaking to the chatbot helped me learn about my surroundings.**	7-point-Likert$^{1}$
*Please indicate what best matches how you feel about the statement.*	
**Q38: Overall, I was successful in gaining a better understanding of my surroundings along the route to the Kunsthaus.**	7-point-Likert$^{1}$
*Please complete this questionnaire by reading each statement carefully and indicate what best matches how you feel about the statement.*	
**Q39: I would engage in a conversation with a** ***voice-based chatbot*** **in a Robotaxi when I am in a** ***familiar*** **environment.**	7-point-Likert$^{1}$
**Q40: I would engage in a conversation with a** ***voice-based chatbot*** **in a Robotaxi when I am in an** ***unfamiliar*** **environment.**	7-point-Likert$^{1}$
*Please complete this questionnaire by reading each statement carefully and indicate what best matches how you feel about the statement.*	
**Q41: The chatbots’ responses matched the personality you defined in the onboarding process.**	7-point-Likert$^{1}$
**Q42: If you had the chance to use the system again. How would you define the personality of the chatbot?**	Text
**Q43: How would you rate your preferred conversation style with such a system?**	7-point-Likert$^{3}$
**Q44: Would you be comfortable sharing personal data to personalize future rides?**	Yes/No
**Q45: Which of the following data would you feel comfortable sharing to personalize future rides?**	Multiple Choice (randomized)
— Chat History/Conversation Transcripts	
— Conversation Preferences (e.g. topics, communication style)	
— Location History (e.g. past destinations, landmark interests, familiar locations)	
— Navigational Preferences (e.g. preferred route)	
**Q46: I was familiar with the area before the simulation.**	7-point-Likert$^{1}$
**Q47: What is your age?**	Number
**Q48: What gender do you identify as?**	Single Choice
**Q49: What is your mother tongue?**	Text

$^{a}$ 1) Strongly Disagree to 7) Strongly Agree

$^{b}$ 1) Very Dissatisfied to 7) Very Satisfied

$^{c}$ 1) Strongly Information-Focused to 7) Strongly Conversation-Focused

## References

[1] Kolodny L (2025) Waymo says it has doubled its weekly paid robotaxi trips to 100,000 since may. https://www.cnbc.com/2024/08/20/waymo-has-doubled-its-weekly-paid-robotaxi-trips-to-100000-since-may.html. Accessed: 25 June 2025

[2] Cheng E (2024) Baidu’s robotaxi unit is exploring expansion into global markets in the “near future”. https://www.cnbc.com/2024/10/09/baidus-robotaxi-unit-is-exploring-expansion-into-global-markets-in-the-near-future.html. Accessed: 25 June 2025

[3] Chiao D et al (2024) The autonomous vehicle industry moving forward | mckinsey. https://www.mckinsey.com/features/mckinsey-center-for-future-mobility/our-insights/autonomous-vehicles-moving-forward-perspectives-from-industry-leaders. Accessed: 25 June 2025

[4] Statista (2020) Autonomous vehicles: a statista dossier plus on self-driving cars. https://www.statista.com/study/69417/autonomous-vehicles/, https://www.statista.com/press/p/autonomous_cars_2020/. Accessed: 25 June 2025

[5] Ribeiro MA, Gursoy D, Chi OH (2022) Customer acceptance of autonomous vehicles in travel and tourism. J Travel Res 61:620–636. 10.1177/0047287521993578

[6] Pakusch C, Meurer J, Tolmie P, Stevens G (2020) Traditional taxis vs automated taxis - Does the driver matter for Millennials? Travel Behaviour and Society 21:214–225 (https://linkinghub.elsevier.com/retrieve/pii/S2214367X20301897)

[7] Dai J, Li R, Liu Z, Lin S (2021) Impacts of the introduction of autonomous taxi on travel behaviors of the experienced user: evidence from a one-year paid taxi service in Guangzhou, China. Transportation Research Part C Emerging Technologies 130:103311 (https://linkinghub.elsevier.com/retrieve/pii/S0968090X21003193)

[8] Le Gallic T, Aguilera A (2022) Anticipating Changes in Lifestyles That Shape Travel Behavior in an Autonomous Vehicle Era–A Method-Oriented Systematic Literature Review. Future Transportation 2:605–624 (https://www.mdpi.com/2673-7590/2/3/33)

[9] Thomopoulos N, Cohen S, Hopkins D, Siegel L, Kimber S (2021) All work and no play? Autonomous vehicles and non-commuting journeys. Transp Rev 41:456–477. 10.1080/01441647.2020.1857460

[10] García-Almeida DJ, Klassen N (2017) The influence of knowledge-based factors on taxi competitiveness at island destinations: an analysis on tips 59:110–122. https://linkinghub.elsevier.com/retrieve/pii/S0261517716301194

[11] Mondschein A, Blumenberg E, Taylor B (2010) Accessibility and Cognition: The Effect of Transport Mode on Spatial Knowledge. Urban Studies 47:845–866 (https://journals.sagepub.com/doi/10.1177/0042098009351186)

[12] Chrastil ER, Warren WH (2013) Active and passive spatial learning in human navigation: acquisition of survey knowledge 39:1520–1537 10.1037/a0032382 23565781

[13] Qin Y, Karimi HA, Harris D, Li W-C (eds) (2020) Spatial knowledge acquisition for cognitive maps in autonomous vehicles. (eds Harris D, Li W-C) Engineering Psychology and Cognitive Ergonomics. Cognition and Design, 384–397 Springer International Publishing, Cham. https://link.springer.com/chapter/10.1007/978-3-030-49183-3_30

[14] Dahmani L, Bohbot VD (2020) Habitual use of GPS negatively impacts spatial memory during self-guided navigation. Sci Rep 10:6310 (https://www.nature.com/articles/s41598-020-62877-0) 32286340 10.1038/s41598-020-62877-0 PMC7156656

[15] Ishikawa T (2019) Satellite Navigation and Geospatial Awareness: Long-Term Effects of Using Navigation Tools on Wayfinding and Spatial Orientation. Prof Geogr 71:197–209. 10.1080/00330124.2018.1479970

[16] Brügger A, Richter K-F, Fabrikant SI (2019) How does navigation system behavior influence human behavior? Cognitive Research Principles and Implications 4:1–5. 10.1186/s41235-019-0156-5 30758681 PMC6374493

[17] Ruginski IT, Creem-Regehr SH, Stefanucci JK, Cashdan E (2019) GPS use negatively affects environmental learning through spatial transformation abilities 64:12–20. https://linkinghub.elsevier.com/retrieve/pii/S0272494418308211

[18] Antrobus V, Large D, Burnett G, Hare C (2019) Enhancing Environmental Engagement with Natural Language Interfaces for In-Vehicle Navigation Systems 72:513–527. 10.1017/S037346331800108X

[19] Liu J, Singh AK, Wunderlich A, Gramann K, Lin C-T (2022) Redesigning navigational aids using virtual global landmarks to improve spatial knowledge retrieval. NPJ Science of Learning 7:1–12 (https://www.nature.com/articles/s41539-022-00132-z) 35853945 10.1038/s41539-022-00132-z PMC9296625

[20] Jarke M, Pasedach K, Pohl K (eds) (1997) Spatial Cognition: The Role of Landmark, Route, and Survey Knowledge in Human and Robot Navigation. Springer, Berlin Heidelberg, Berlin, Heidelberg

[21] Schwering A, Krukar J, Li R, Anacta VJ, Fuest S (2017) Wayfinding Through Orientation. Spatial Cognition & Computation 17:273–303. 10.1080/13875868.2017.1322597

[22] Rege A, Currano R, Sirkin D, Kim E (2024) ‘Talking with your Car’: Design of Human-Centered Conversational AI in Autonomous Vehicles, 338–349 ACM. https://dl.acm.org/doi/10.1145/3640792.3675713

[23] Acer UG, Van Den Broeck M, Kawsar F (2019) On Hyper-local Conversational Agents in Urban Settings, 1–4 ACM. https://dl.acm.org/doi/10.1145/3325426.3329949

[24] Ishikawa T (2021) Spatial thinking, cognitive mapping, and spatial awareness 22:89–96. 10.1007/s10339-021-01046-1 34313882

[25] Afrooz A, White D, Parolin B (2018) Effects of active and passive exploration of the built environment on memory during wayfinding 101:68–74. https://linkinghub.elsevier.com/retrieve/pii/S0143622817312675

[26] Cohen PR, Oviatt S (2017) in Multimodal speech and pen interfaces (eds Oviatt S et al) The Handbook of Multimodal-Multisensor Interfaces: Foundations, User Modeling, and Common Modality Combinations - Vol 1 403–447 ACM. http://dl.acm.org/citation.cfm?id=3015795

[27] Oviatt S, Cohen PR The Paradigm Shift to Multimodality in Contemporary Computer Interfaces Synthesis Lectures on Human-Centered Informatics (Springer International Publishing). https://link.springer.com/10.1007/978-3-031-02213-5

[28] Serrano M, Juras D, Nigay L (2008) A three-dimensional characterization space of software components for rapidly developing multimodal interfaces, ICMI ’08, 149–156 (Association for Computing Machinery). https://dl.acm.org/doi/10.1145/1452392.1452421

[29] Signer B (2024) in Pen-Based Interaction 1–22 (Springer, Cham). https://link.springer.com/rwe/10.1007/978-3-319-27648-9_102-1

[30] Reeves LM et al (2004) Guidelines for multimodal user interface design 47:57–59. https://dl.acm.org/doi/10.1145/962081.962106

[31] Flohr LA, Kalinke S, Krüger A, Wallach DP (2021) Chat or Tap? - Comparing Chatbots with ‘Classic’ Graphical User Interfaces for Mobile Interaction with Autonomous Mobility-on-Demand Systems, 1–13 ACM. https://dl.acm.org/doi/10.1145/3447526.3472036

[32] Committee O-RADO (2021). Taxonomy and Definitions for Terms Related to Driving Automation Systems for On-Road Motor Vehicles. 10.4271/J3016_202104

[33] Yao X, Liu J, Wang X, Xiao Y, Qi G (2025) Transition toward driverless robotaxi: Role of social anxiety, perceived safety, and travel habit. Transport Res F: Traffic Psychol Behav 109:1402–1418. 10.1016/j.trf.2025.01.031

[34] Schuß M, Manger C, Löcken A, Riener A (2022) You’ll Never Ride Alone: Insights into Women’s Security Needs in Shared Automated Vehicles, 13–23 ACM. https://dl.acm.org/doi/10.1145/3543174.3546848

[35] Yin H, Cherchi E (2024) Willingness to pay for automated taxis: a stated choice experiment to measure the impact of in-vehicle features and customer reviews 51:51–72. https://link.springer.com/10.1007/s11116-022-10319-3

[36] Qiu X (2020) Impact of Learning Methods on Spatial Knowledge Acquisition Frontiers in Psychology 11 10.3389/fpsyg.2020.01322 PMC7308434 32612561

[37] Chrastil ER, Warren WH (2015) Active and passive spatial learning in human navigation: acquisition of graph knowledge. J Exp Psychol Learn Mem Cogn 41:1162–1178 25419818 10.1037/xlm0000082

[38] Perrine KA, Kockelman KM, Huang Y (2020) Anticipating long-distance travel shifts due to self-driving vehicles 82:102547. https://linkinghub.elsevier.com/retrieve/pii/S0966692318306367

[39] Powell A, Research HoU, Design Driven by Waymo, Designed with Trust. https://waymo.com/blog/2019/08/driven-by-waymo-designed-with-trust

[40] Brinkley J, Huff EW, Gluck A, Enam MA (2024) An Autoethnographic Study of the Waymo One Autonomous Ridesharing Ecosystem: Exploring Issues of Accessibility, 1–6. https://ieeexplore.ieee.org/document/10555855

[41] Braun M, Broy N, Pfleging B, Alt F (2019) Visualizing natural language interaction for conversational in-vehicle information systems to minimize driver distraction. Journal on Multimodal User Interfaces 13:71–88. 10.1007/s12193-019-00301-2

[42] Lugano G (2017) Virtual assistants and self-driving cars, 1–5. https://ieeexplore.ieee.org/abstract/document/7972192

[43] Tang Y, Guo P, Tang CS, Wang Y (2021) Gender-Related Operational Issues Arising from On-Demand Ride-Hailing Platforms: Safety Concerns and System Configuration 30:3481–3496. 10.1111/poms.13444

[44] Alpers BS et al (2020) Capturing Passenger Experience in a Ride-Sharing Autonomous Vehicle: The Role of Digital Assistants in User Interface Design, 83–93 ACM. https://dl.acm.org/doi/10.1145/3409120.3410639

[45] Ait Baha T, El Hajji M, Es-Saady Y, Fadili H (2023) The power of personalization: a systematic review of personality-adaptive chatbots. SN Computer Science 4:661. 10.1007/s42979-023-02092-6

[46] Seaborn K, Miyake NP, Pennefather P, Otake-Matsuura M (2021) Voice in human-agent interaction: a survey. ACM Comput Surv 54(4):1–43. 10.1145/3386867

[47] Lee S, Ratan R, Park T (2019) The Voice Makes the Car: enhancing Autonomous Vehicle Perceptions and Adoption Intention through Voice Agent Gender and Style 3:20. https://www.mdpi.com/2414-4088/3/1/20

[48] Brandt A, Hazel S (2024) Towards interculturally adaptive conversational AI. Applied Linguistics Review. 10.1515/applirev-2024-0187

[49] Shumanov M, Johnson L (2021) Making conversations with chatbots more personalized. Comput Hum Behav 117:106627 (https://linkinghub.elsevier.com/retrieve/pii/S0747563220303745)

[50] Cheverst K, Davies N, Mitchell K, Friday A, Efstratiou C (2000) Developing a context-aware electronic tourist guide: Some issues and experiences, 17–24 (ACM). https://dl.acm.org/doi/10.1145/332040.332047

[51] Schöning J, Hecht B, Starosielski N (2008) Evaluating automatically generated location-based stories for tourists, CHI EA ’08, 2937–2942 (Association for Computing Machinery, New York, NY. USA. 10.1145/1358628.1358787

[52] Belz JH et al (2024) Story-Driven: Exploring the Impact of Providing Real-time Context Information on Automated Storytelling, UIST ’24, 1–15 (Association for Computing Machinery). https://dl.acm.org/doi/10.1145/3654777.3676372

[53] OpenAI (2025) OpenAI Platform. https://platform.openai.com. Accessed: 25 June 2025

[54] OpenStreetMap contributors (2025) OpenStreetMap Wiki and Maps. Planet dump retrieved from https://planet.osm.org. https://www.openstreetmap.org. Accessed: 25 June 2025

[55] Wikimedia Foundation (2025) Mediawiki. https://www.mediawiki.org/wiki/MediaWiki. Accessed: 25 June 2025

[56] Mapbox (2025) Mapbox Maps. https://www.mapbox.com/about/maps. Accessed: 25 June 2025

[57] Richardson AE, Montello DR, Hegarty M (1999) Spatial knowledge acquisition from maps and from navigation in real and virtual environments 27:741–750. 10.3758/BF03211566 10479831

[58] Delikostidis I, van Elzakker CP, Kraak M-J (2016) Overcoming challenges in developing more usable pedestrian navigation systems. Cartogr Geogr Inf Sci 43:189–207. 10.1080/15230406.2015.1031180

[59] Google Developers (2025) Place types. https://developers.google.com/maps/documentation/places/web-service/place-types. Accessed: 25 June 2025

[60] Vaittinen T, McGookin D (2016) Phases of urban tourists’ exploratory navigation: a field study, DIS ’16, 1111–1122 ACM. 10.1145/2901790.2901795

[61] Hart SG, Staveland LE (1988) in Development of nasa-tlx (task load index): Results of empirical and theoretical research (eds Hancock PA, Meshkati N) Human Mental Workload, Vol 52 of Advances in Psychology 139–183 North-Holland,. 10.1016/S0166-4115(08)62386-9

[62] Schrepp M, Thomaschewski J, Hinderks A (2017) Design and evaluation of a short version of the user experience questionnaire (ueq-s). International Journal of Interactive Multimedia and Artificial Intelligence 4:103–108 (http://www.ijimai.org/journal/sites/default/files/files/2017/09/ijimai20174_6_14_pdf_20309.pdf)

[63] Holmes S et al (2019) Usability testing of a healthcare chatbot: Can we use conventional methods to assess conversational user interfaces?, ECCE ’19, 207–214 (Association for Computing Machinery, New York, NY. USA. 10.1145/3335082.3335094

[64] Borsci S et al (2022) The Chatbot Usability Scale: The Design and Pilot of a Usability Scale for Interaction with AI-Based Conversational Agents, 26:95–119. 10.1007/s00779-021-01582-9

[65] Mildner T et al (2024) Listening to the voices: Describing ethical caveats of conversational user interfaces according to experts and frequent users, CHI ’24 (Association for Computing Machinery, New York, NY. USA. 10.1145/3613904.3642542

[66] Park SY, Moore DJ, Sirkin D (2020) What a Driver Wants: User Preferences in Semi-Autonomous Vehicle Decision-Making. Proceedings of the 2020 CHI Conference on Human Factors in Computing Systems 1–13. https://dl.acm.org/doi/10.1145/3313831.3376644

[67] Kim H, Lee I-K (2025) In-vehicle full-window augmented reality system (FARS) for passengers: effects on spatial knowledge and user experience. Virtual Reality 29:34. 10.1007/s10055-024-01068-y

[68] Schade E (2026) RideGuide: multimodal conversational tour guide for passenger engagement and spatial learning in robotaxis. Zenodo. 10.5281/zenodo.15699734

